# Hormopriming to Mitigate Abiotic Stress Effects: A Case Study of *N*^9^-Substituted Cytokinin Derivatives With a Fluorinated Carbohydrate Moiety

**DOI:** 10.3389/fpls.2020.599228

**Published:** 2020-12-10

**Authors:** Magdaléna Bryksová, Andrea Hybenová, Alba E. Hernándiz, Ondřej Novák, Aleš Pěnčík, Lukáš Spíchal, Nuria De Diego, Karel Doležal

**Affiliations:** ^1^Department of Chemical Biology and Genetics, Centre of the Region Haná for Biotechnological and Agricultural Research, Faculty of Science, Palacký University, Olomouc, Czechia; ^2^Laboratory of Growth Regulators, Palacký University and Institute of Experimental Botany, Czech Academy of Sciences, Olomouc, Czechia

**Keywords:** abiotic stress, antisenescence, Arabidopsis, cytokinin analogs, hormopriming, plant biostimulant characterization index

## Abstract

Drought and salinity reduce seed germination, seedling emergence, and early seedling establishment, affect plant metabolism, and hence, reduce crop yield. Development of technologies that can increase plant tolerance of these challenging growth conditions is a major current interest among plant scientists and breeders. Seed priming has become established as one of the practical approaches that can alleviate the negative impact of many environmental stresses and improve the germination and overall performance of crops. Hormopriming using different plant growth regulators has been widely demonstrated as effective, but information about using cytokinins (CKs) as priming agents is limited to only a few studies using kinetin or 6-benzylaminopurine (BAP). Moreover, the mode of action of these compounds in improving seed and plant fitness through priming has not yet been studied. For many years, BAP has been one of the CKs most commonly applied exogenously to plants to delay senescence and reduce the impact of stress. However, rapid endogenous *N*^9^-glucosylation of BAP can result in negative effects. This can be suppressed by hydroxylation of the benzyl ring or by appropriate *N*^9^ purine substitution. Replacement of the 2′ or 3′ hydroxyl groups of a nucleoside with a fluorine atom has shown promising results in drug research and biochemistry as a means of enhancing biological activity and increasing chemical or metabolic stability. Here, we show that the application of this chemical modification in four new *N*^9^-substituted CK derivatives with a fluorinated carbohydrate moiety improved the antisenescence properties of CKs. Besides, detailed phenotypical analysis of the growth and development of Arabidopsis plants primed with the new CK analogs over a broad concentration range and under various environmental conditions revealed that they improve growth regulation and antistress activity. Seed priming with, for example, 6-(3-hydroxybenzylamino)-2′-deoxy-2′-fluoro-9-(β)-D-arabinofuranosylpurine promoted plant growth under control conditions and alleviated the negative effects of the salt and osmotic stress. The mode of action of this hormopriming and its effect on plant metabolism were further analyzed through quantification of the endogenous levels of phytohormones such as CKs, auxins and abscisic acid, and the results are discussed.

## Introduction

Global climate change is increasing the severity of drought and soil salinity, with deleterious effects on already-stressed agricultural ecosystems. Moreover, predictions for the future indicate that the areas affected by these two types of stress are going to expand and as a consequence the productivity of many plant species will be reduced (Savvides et al., 2016; Uddin et al., 2016; Pavlů et al., 2018). The development of biotechnological approaches that increase plant tolerance and assure the maintenance of yield under these challenging growth conditions is therefore one of the main aims of plant scientists and breeders.

One of the technologies that attracts a high level of interest nowadays is “seed priming” (Savvides et al., 2016). Seed priming is an effective pre-sowing technology in which seeds are treated with small doses of certain agents just prior to germination. Unlike un-primed seeds, primed seeds are able to respond to very low levels of specific stimuli, which helps plants to prepare their metabolism for better defense responses to stress factors (Conrath, 2011; Paparella et al., 2015). Thus, priming can improve seed performance, ensure higher uniformity among the seeds, result in faster and better synchronized germination, and enhance plant growth (Gamir et al., 2014; Ibrahim, 2016; Lutts et al., 2016). Several methods of seed priming, including hydropriming, osmopriming, hormopriming, biopriming, and chemical priming, have been developed (Jisha et al., 2013; Paparella et al., 2015). Hormopriming consists in the exogenous application of plant growth regulators or phytohormones that can stimulate seed imbibition and modify seed metabolism. The plant growth regulators most often used in this way are abscisic acid (ABA), gibberellins, cytokinins (CKs), auxins, ethylene, and polyamines (reviewed by De Diego and Spíchal, 2020).

In plants, CKs are involved in many biological processes: regulating sink/source relationships (Roitsch and Ehneß, 2000; Werner et al., 2008), nutrient uptake (Sakakibara, 2006; Criado et al., 2009), leaf senescence (Jordi et al., 2000; Marchetti et al., 2018), and responses to abiotic stress (Bielach et al., 2017). Since the discovery of the first CK, kinetin, by Skoog, Miller, and associates in 1955 (Miller et al., 1955), the number of chemicals fitting the definition of CK has grown to include a large array of natural and synthetic compounds, among which are adenine and phenylurea derivatives (Mok and Mok, 2001). Depending on their chemical structure, natural CKs are adenine derivatives with an isoprenoid or aromatic *N*^6^-side chain (Mok and Mok, 2001). CKs are present in plants in the forms of free bases, glucosides, nucleosides and nucleotides, at very low concentrations [pmol g^–1^ fresh weight (FW)] (Strnad, 1997). The precursor nucleotides, namely *N*^9^-riboside-5′- mono-, di-, and tri-phosphates, are endogenously synthesized *de novo* and converted to active free bases. The bases can be subsequently conjugated with glucose at positions *N*^3^, *N*^7^, and *N*^9^ of the purine ring and at the hydroxyl group of the side chain, which can be also conjugated with xylose (Frébort et al., 2011). Addition of sugar moieties to the *N*^9^ position of the purine ring can also form *N*^9^-riboside-glucoside (George et al., 2008). While the *O*-glycosylated forms can be converted back into active CKs, *N*-glycosylation occurs primarily at positions *N*^7^ or *N*^9^ of the purine ring, and is thought to be irreversible (Brzobohatý et al., 1993), except in the case of the *t*Z forms (Hošek et al., 2020). Furthermore, it has been demonstrated that some of these conjugates may have significant CK activity, especially in the case of *N*^9^-riboside analogs (Doležal et al., 2007).

The aromatic CK benzylaminopurine (BAP) is considered the most effective and the cheapest CK, which has led to its widespread use in biotechnology. However, many disadvantages associated with its applications have been reported (Werbrouck et al., 1995; Bairu et al., 2009). Negative effects can be caused by natural *N*^9^-glucosylation of the applied purine based CK, leading to extensive accumulation of non-active CK glucosides (Werbrouck et al., 1995). Moreover, *N*^9^-glucosides can activate ethylene production and the ethylene signaling pathway causing inhibition of root elongation (Podlešáková et al., 2012). One way of avoiding the negative effects of *N*^9^-glucosylation is to suppress it by appropriate *N*^9^ purine substitution in BAP or by hydroxylation of its benzyl ring (Plíhal et al., 2013).

Fluorination has a long tradition in nucleoside chemistry and the replacement of the 2′ or 3′ hydroxyl group of a nucleoside with a fluorine atom causes only a minor change in the overall structure, but significantly affects the stereoelectronic properties of the sugar moiety (Thibaudeau et al., 1998). It has been reported that important factors in the substitution of fluorine for hydrogen are the comparable size of the two atoms and the powerful electron withdrawing properties of fluorine relative to hydrogen, as well as the increased stability of the carbon-fluorine bond relative to the carbon-hydrogen bond. Hence, replacement of hydrogen by fluorine in a bioactive molecule is expected to cause minimal steric perturbations with respect to the molecule’s mode of binding to receptors or enzymes (Pitzer, 1960). Moreover, replacement of the hydrogen by fluorine causes not only changes in biological activity, but also increases the chemical and metabolic stability of nucleosides. The conformation of the sugar moiety of these analogs is strongly affected by the presence of the fluorine substituent and is different from that of natural deoxynucleosides (Pankiewicz et al., 1992).

Nucleosides bearing fluorine or fluorinated substituents within the carbohydrate moiety have been widely used in biochemical research and therapeutic treatment (Meng and Qing, 2006; Hagmann, 2008; Kirk, 2008). However, to date only few fluorinated CK derivatives have been prepared and their biological activity tested in plants (Clemenceau et al., 1996; Doležal et al., 2007). Only recently, several 6-benzylaminopurines substituted with β-D-arabinose at the *N*^9^-position with similar structures were synthesized in our laboratory, and subsequently patented as powerful antisenescence compounds compared with BAP, but the activity of these compounds were only tested in a detached wheat leaves senescence bioassay (Patent No. US 10,100,077 B2, 2018). Here, we present a new class of *N*^6^-substituted-2′-deoxy-2′-fluoro-9-(β)-D-arabinofuranosylpurine derivatives which show not only high levels of antisenescence activity but also promise as seed priming agents due to their high efficiency as plant growth promotors and plant stress alleviators. Their mode of action as priming agents is also discussed.

## Materials and Methods

### General Synthesis of *N*^6^-Substituted-2′-Deoxy-2′-Fluoro-9-(β)-D-Arabinofuranosylpurine Derivatives

All the compounds presented here were prepared by a slightly modified one-step synthesis (Wan et al., 2005) of 9-(2′-deoxy-2′-fluoro-β-D-arabinofuranosyl)hypoxanthine with benzylamine or isopentenylamine hydrochloride as appropriate in the presence of BOP and DIPEA in DMF (Figure 2). Firstly 9-(2′-deoxy-2′-fluoro-β-D-arabinofuranosyl)hypoxanthine (200 mg, 1 equiv.) and BOP (396 mg, 1.2 equiv.) were mixed together in DMF (4 mL) and subsequently DIPEA (194 μL, 1.5 equiv.) and benzylamine (1–3) or isopentenylamine hydrochloride (4) (1.2 equiv.) as the last component were added. Each reaction mixture was stirred under an argon atmosphere in an oil bath at a temperature of 55–60°C for 24 h and the effectiveness of the reaction was checked by Thin-layer chromatography (TLC) (CHCl_3_/MeOH 4:1). The reaction mixture was evaporated using a vacuum rotary evaporator to give a specifically colored gel. The resulting residue was carefully purified by column chromatography (1 and 3) or by preparative HPLC (2 and 4) to give the desired product, which in some cases (1 and 3) could be crystallized from various solvents.

### General Procedures

The chromatographic purity and mass spectra of the compounds described were characterized using the HPLC-PDA-MS method. Samples (10 μL of 3 × 10^–5^ M in 1% methanol) were injected onto a reverse-phased column (Symmetry C18, 5 μm, 150 mm × 2.1 mm; Waters, Milford, MA, United States) tempered at 25°C. Solvent (A) consisted of 15 mM ammonium formate adjusted to pH 4.0 and solvent (B) consisted of methanol. The flow-rate was set to 200 μL min^–1^. A binary gradient was used: 0 min, 10% of B; 24 min; 90% of B; 34 min; 90% of B; 45 min; 10% of B using a Waters Alliance 2695 Separations Module (Waters, Manchester, United Kingdom). Then the effluent was introduced to a Waters 2996 PDA detector (Waters, Manchester, United Kingdom) (scanning range 210–700 nm with 1.2 nm resolution) and a tandem mass analyzer Q-Tof micro Mass Spectrometer (Waters, Manchester, United Kingdom) with an electrospray. The cone voltage was set to 20 V. Exact mass was determined by QTOF-MS (Synapt G2-Si, Waters, United Kingdom) operating in positive ion mode and recorded as (M + H)^+^. Melting points were determined on a Büchi Melting Point B-540 apparatus and are uncorrected. ^1^H NMR spectra were analyzed on a Jeol 500 SS spectrometer operating at a temperature of 300 K and a frequency of 500.13 MHz. The samples were prepared by dissolving in DMSO-d_6_. Tetramethylsilane (TMS) was used as an internal standard. TLC was carried out using silica gel 60 WF_254_ plates (Merck). Purification *via* column chromatography was performed using silica gel Davisil R LC60A 40-63 micron.

### HPLC-MS Purification

A preparative HPLC-MS chromatography machine (Agilent 1290 Infinity II) was used coupled to a UV-VIS detector with a mass LC/MSD detector (Agilent InfinityLab) and an Agilent Prep-C18 column (5 μm, 21.2 mm × 50 mm, Waters, Milford, MA, United States) to obtain the final products. Analyzed samples were dissolved in 50% MeOH before injection. The mobile phase was methanol (A):H_2_O (B) with a flow rate of 20 mL min^–1^ and linear gradients (0 min, 10% B; 0–12 min; 90% B) were used.

### HRMS Conditions

Samples (5 μL) were characterized using the HPLC-PDA-MS method. They were injected onto a reversed-phase column (Symmetry C18, 5 μm, 150 mm × 2.1 mm; Waters, Milford, MA, United States) incubated at 40°C. Solvent A was 15 mM ammonium formate adjusted to pH 4.0. Solvent B was methanol. The following linear gradient was used at a flow rate of 250 μL min^–1^: 0 min, 10% B; 0–15 min, 90% B. The effluent was introduced to a DAD detector (scanning range 210–400 nm with 1.2 nm resolution) and then to an electrospray source (source temperature 150°C, desolvation temperature 550°C, capillary voltage 1 kV, cone voltage 25 V). Nitrogen was used as the cone gas (50 L h^–1^) and the desolvation gas (1000 L h^–1^). Data acquisition was performed in full-scan mode (50–1000 Da) with a scan time of 0.5 s and collision energy of 4 eV; argon was used as the collision gas (optimized pressure of 5 × 10^–3^ mbar). Analyses were performed in positive mode (ESI^+^), therefore protonated molecules (M + H)^+^ were collected in each MS spectrum. For exact mass determination experiments, external calibration was performed using lock spray technology and a mixture of leucine/encephalin (50 pg μL^–1^) in an acetonitrile and water (1:1) solution with 0.1% formic acid as a reference. Accurate masses were calculated and used to determine the elemental composition of the analytes with a fidelity better than 1.0 ppm.

### Cytokinin Bioassays

Cytokinin bioassays, including Amaranthus, tobacco callus and senescence bioassays, were carried out as previously described by Holub et al. (1998), using BAP as a positive control for all three classical CK bioassays. Results were recorded to define the highest activities of the four compounds prepared. All of them were dissolved in 0.5% DMSO and tested at five concentrations (from 10^–8^ to 10^–4^ M).

### Plant Phenotyping – Rosette Growth of Seedlings From Arabidopsis Hormoprimed Seeds

The four compounds synthesized were tested as priming agents under optimal and two different stress conditions. Arabidopsis seeds (*Arabidopsis thaliana* accession Col-0) were sterilized and germinated as described by Ugena et al. (2018). During germination the compounds were added at four different concentrations (from 10^–7^ to 10^–4^ M) to germination medium containing 0.5× MS (pH 5.7) supplemented with a gelling agent (0.6% Phytagel; Sigma-Aldrich, Germany). Three days after germination, seedlings of similar size were transferred under sterile conditions into 48-well plates (Jetbiofil, Guangzhou, China). One seedling was transferred to each well filled with 850 mL 1× MS medium (pH 5.7; supplemented with 0.6% Phytagel), without stress treatment (optimal conditions) or containing 100 mM NaCl (as salt stress) or 100 mM mannitol (as osmotic stress), and the plates were sealed with perforated transparent foil allowing gas and water exchange. Hormopriming of 10^–8^ M BAP was also used as positive control for all tested growth conditions.

The 48-well plates containing the transferred Arabidopsis seedlings were placed in an OloPhen platform,^1^ which uses the PlantScreen^TM^ XYZ system installed in a growth chamber with a controlled environment, and cool-white LED and far-red LED lighting (Photon Systems Instruments, Brno, Czechia). The conditions were set to simulate a long day with a temperature regime of 22/20°C in a 16/8 h light/dark cycle, an irradiance of 120 μmol photons of PAR m^2^ s^–1^ and a relative humidity of 60%. The PlantScreen^TM^ XYZ system consists of a robotically driven arm holding an RGB camera with customized lighting panel and growing tables. The XYZ robotic arm was automatically moved above the plates to take RGB images of single plates from the top. The imaging of each 48 well plate was performed twice per day (at 10 a.m. and 4 p.m.) for 7 days as described in Ugena et al. (2018). As outcome, the individual image of 48 Arabidopsis seedlings per variant (treatment vs. growth condition) as biological replicates were used for the analyzed phenotyping traits.

Different traits were determined from the RGB images: Arabidopsis rosette growth curves [as changes in the green area (Pixels)], relative (RGR) and absolute (AGR) growth rate and final rosette size. All these traits were then used to define the mode of action of the compound under test. Using the traits, the plant biostimulant characterization (PBC) index was determined as described by Ugena et al. (2018). The PBC index was calculated as the sum of the values obtained from each phenotyping trait calculated as the differences (as the log2 of the ratio in each case) between the controls and treatment variants (compound and concentration) under the same growth conditions.

### Determination of Arabidopsis Rosette Color Indices

To estimate the greenness of the Arabidopsis seedlings, and changes in leaf color, three vegetation indices (NGRDI, VARI, and GLI), which have been shown to be correlated with plant biomass, nutrient status, or tolerance to abiotic stress (Gitelson et al., 2002; Perry and Roberts, 2008; Hunt et al., 2013), were used. The images captured on the seventh day of an Arabidopsis rosette growth assay subjected to HTS were segmented for the extraction of leaf rosettes using software described in our previous report (De Diego et al., 2017). The values corresponding to particular color channels (red, R; green, G; and blue, B) were then extracted for each pixel within the plant mask, and the vegetation indices were calculated as described by Ugena et al. (2018). Subsequently, indices representing particular seedlings were determined by calculating the mean values for each plant mask. The mean and the standard error (SE) values for each 48-well plate were then calculated and represented in a graph.

### Plant Hormone Quantification

Four independent biological replicates consisted in four individual pools from 12 Arabidopsis seedlings per variant were collected for the hormonal analysis. After purification and extraction, the concentration of each analyte was calculated using the standard isotope dilution method (Rittenberg and Foster, 1940). Briefly, as the first step a micro solid-phase extraction (μSPE) based on StageTip (STop And Go Extraction Tip) technology was used to purify the plant tissue samples. The μSPE protocol used in CK extraction and purification was applied as described by Svačinová et al. (2012), whereas auxins and ABA were isolated as described by Pênčík et al. (2018). CKs were determined using ultra-high performance liquid chromatography-electrospray tandem mass spectrometry (an Acquity UPLC I-Class System coupled with a Xevo TQ-S MS, Waters). Quantification of auxins and ABA was performed and the concentration of each analyte was calculated using the standard isotope dilution method on a 1260 Infinity II system coupled with a 6495B Triple Quadrupole LC/MS system (Agilent Technologies).

### Statistical Analysis

To assess differences between treatments (compound and concentration) values for each non-invasive trait extracted by means of image analysis, a non-parametric (Dunn’s test after Kruskal–Wallis’ test parametric) method and a parametric method (Tukey’s HSD test after two-way ANOVA) were applied using the packages *multcomp, FSA*, and *agricolae* in RStudio (Version 1.1.463 – 2009-2018 RStudio, Inc.). Multivariate statistical analyses, including heatmap and principal component (PC) analysis, were also performed in RStudio using the packages *gplots*, *cluster*, *tidyverse*, *factoextra*, *heatmap.plus*, *ggpubr*, *factoextra*, *FactoMineR*, and *corrplot*.

## Results

### Synthesis of Four *N*^9^-Substituted CK Derivatives With a Fluorinated Carbohydrate Moiety

**FIGURE 1:**
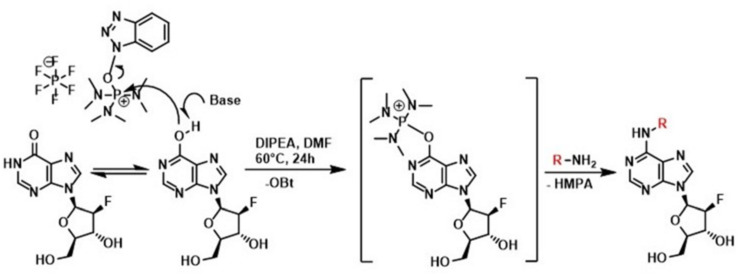
Scheme for the preparation of substituted 6-benzylamino-2′-deoxy-2-′fluoro-9-(β)-D-arabinofuranosylpurine derivatives by a method using Castro’s reagent and Hunig base.

In this work, a group of three *N*^9^-substituted aromatic or one isoprenoid CK derivatives with a fluorinated carbohydrate moiety were synthesized (Figure 1) and their biological activity was investigated. The compounds prepared were characterized by ^1^H NMR, elemental analysis, melting points, TLC and ESI + MS. The purity of the prepared derivatives was confirmed by high-performance liquid chromatography (HPLC-UV) (Table 1).

**FIGURE 2:**
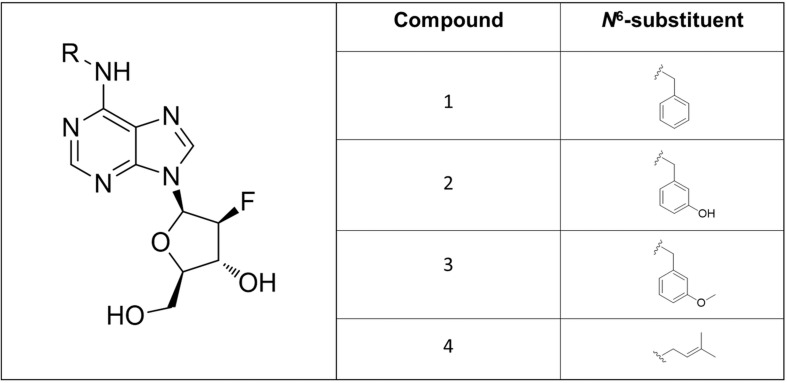
Structures of the newly synthesized 6-benzylamino-2′-deoxy-2-′fluoro-9-(β)-D-arabinofuranosylpurine derivatives.

**TABLE 1:** Physico-chemical properties of four new synthesized compounds: elemental analysis, melting temperature, mass of positively charged molecular ions analyzes by HPLC-MS, purity, and HRMS mass analysis.

Compound	Elemental analysis calculated/found			Mp (°C)	ES-MS (M + H^+^)	HPLC (%)	HRMS			
	% of C	% of H	% of N				Exact mass	Theoretical monoisotopic mass	Difference (ppm)	Elementary analysis (M + H^+^)
1	56.82/56.18	5.05/5.02	19.49/19.28	179–180	360	99^+^	359.1393	359.1394	−0.23	C17H_19_FN_5_O_3_
2	54.40/53.61	4.83/5.00	18.66/18.23	101–103	376	99^+^	375.1341	375.1343	−0.48	C_17_H_19_FN_5_O_4_
3	55.52/56.05	5.18/4.86	17.99/19.24	212–213	390	99^+^	389.1502	389.1499	0.74	C_18_H_21_FN_5_O_4_
4	53.40/54.94	5.98/5.80	20.76/18.73		338	99^+^	337.1552	337.155	0.4	C_15_H_21_FN_5_O_3_

First, the synthesis of a 2-fluoropentose from a pentoside precursor followed by its conversion into 9-(2-deoxy-2-fluoro-β-D-arabinofuranosyl) adenine was performed as reported in 1969 (Wright et al., 1969). Subsequently, 3-deoxy-3-fluoro-D-glucose was synthesized and converted into 2-deoxy-2-fluoro-D-arabinose via oxidation by sodium metaperiodate as described by Reichman et al. (1975). The compound 9-(2-deoxy-2-fluoro-β-D-arabinofuranosyl) adenine (F-ara-A) has previously been prepared by condensation of 6-chloropurine with 2-deoxy-2-fluoro-D-arabinofuranosyl bromide followed by conversion of the purine into adenine, but the reaction produced a mixture of four isomers and only a very low yield of the desired isomer could be isolated (Marquez et al., 1990). Later, a three-step synthesis of 9-(2-deoxy-2-fluoro-β-D-arabinofuranosyl)adenine was carried out via displacement of the 2′-hydroxyl group of 03′,05′,*N*^6^-tritrityladenosine and 03′,05′-ditritylinosine with diethylaminosulfur trifluoride, as published by Pankiewicz et al. (1992). The importance of introducing a fluorine at the 2′(S)(ara) site of purine deoxynucleosides has been highlighted, since 2′-deoxy-2′-fluoroarabinosides have been found to be biologically active and chemically stable against hydrolysis catalyzed both chemically and by purine nucleoside phosphorylase (Chu et al., 1989).

In the present study, all the aforementioned steps were followed and finally the synthesis of new compounds was performed as previously reported by Wan et al. (2005) with some modifications. Typically, the synthesis of purine nucleosides is based on the protection of hydroxyl groups, which prolongs this method to a four-step process with low yield. This transformation usually causes cleavage of the glycosyl bond, therefore only acid-labile protecting groups must be used. In our new simple one-step unprotected synthesis, BOP was used to activate the formation of a C-N bond. Subsequently, substitution by appropriate amines led to the formation of final products, after elimination of hexamethylphosphoramide (HMPA). However, the nucleophilic substitution of unprotected purine nucleosides with amines required longer reaction times compared with their protected counterparts (Wan et al., 2005).

### CK-Like Activity of the New *N*^9^-Substituted CK Derivatives With a Fluorinated Carbohydrate Moiety in Cytokinin Bioassays

To evaluate the CK activities of the newly synthesized compounds, three classical *in vitro* CK bioassays were used. Despite the fact that all four of the new compounds are derived from CKs with known high levels of activity in all three bioassays, their 2′-deoxy-2′-fluoro-9-(β)-D-arabinofuranosyl purine derivatives showed decreases in activity in the Amaranthus and tobacco callus bioassays (Supplementary Table 1). On the other hand, high antisenescence activity was recorded in the bioassay based on evaluating the effect of the compound on retention of chlorophyll in excised wheat leaves kept in the dark (Table 2). The greatest ability to prevent chlorophyll degradation was shown by compounds 1 and 2, which reached, respectively, 277 and 267% of the values for the positive control BAP at concentrations of 10^–4^ M, followed by compound 3, which showed 179% of the BAP activity. Compound 4 had comparable activity to BAP (Table 2). Overall, these results showed that substitution at the *N*^9^ position with a fluorinated carbohydrate moiety selectively influences the CK-like activity, specifically improving the antisenescence properties of CKs modified in this way. This suggests that such CK analogs could activate plant processes related to stress responses and would therefore have antistress properties when applied to plants.

**TABLE 2:** Relative CK activities of four new synthesized compounds in the senescence bioassay.

Compounds	Senescence bioassay	
	Optimal concentration (M)	Relative activity (%)
1	10^–4^	277 (±9)
2	10^–4^	267 (±17)
3	10^–4^	179 (±3)
4	10^–4^	95 (±6)

*The optimal concentration for compounds 1–3 was compared with the activity of benzylaminopurine (BAP), where 100% means 10^–4^ M BAP. The optimal concentration for compound 4 was compared with the activity of isopentenyladenine (iP), where 100% means 10^–4^ M iP.*

### Priming With *N*^9^-Substituted CK Derivatives With a Fluorinated Carbohydrate Moiety Improves the Growth of Arabidopsis Under Both Optimal and Stress Conditions

**FIGURE 3:**
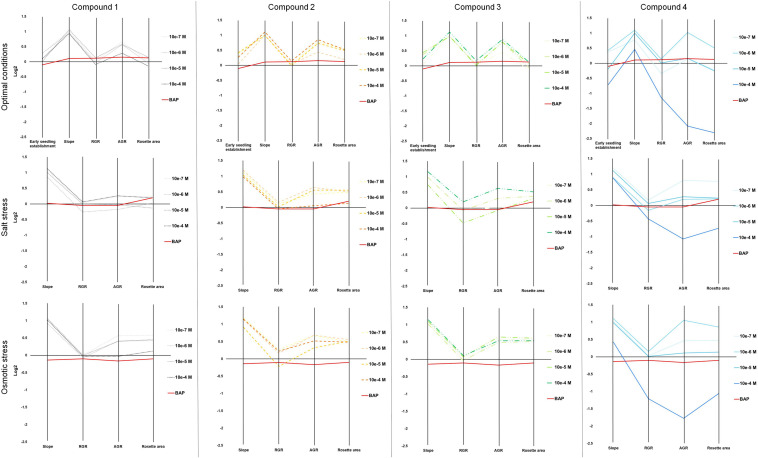
Parallel coordinate plot of the traits (Early seedling establishment, slope of the growing curve, RGR, AGR and the final rosette area) obtained from multi-trait high-throughput screening of Arabidopsis seedlings non-primed (MOCK) or primed with four different *N*^9^-substituted CK derivatives with a fluorinated carbohydrate moiety at four concentrations (10^–7^, 10^–6^, 10^–5^, and 10^–4^ M) and grown under optimal (upper panels), or salt (100 mM NaCl, middle panels) or osmotic (100 mM mannitol, bottom panels) stress conditions (*N* = 48). BAP at 10^–8^ M was used as positive control.

To corroborate the involvement of these compounds in plant stress tolerance and better define the mode of action of our four newly synthesized compounds, we tested their effects on Arabidopsis growth and development under optimal and stress conditions using a complex multi-trait high-throughput screening approach (Ugena et al., 2018). The four compounds were used as seed priming agents at four concentrations (from 10^–7^ to 10^–4^ M). Non-primed and primed seeds were germinated under optimal conditions and then the seedlings were transferred into 48 well plates with 1× MS alone, or supplemented with 100 mM NaCl or 100 mM mannitol to induce salt or osmotic stress, respectively. First, we evaluated how the priming affected early seedling establishment. To do so, the rosette area of the seedlings transferred to control conditions (1× MS) at day 1 was determined. Here, we saw a clear interaction between compound and concentration affecting early seedling establishment (Figure 3 and Supplementary Figure 1). The seedlings developed from seeds primed with all the compounds except compound 1 had increased rosette area. The largest rosettes were observed after priming with the highest concentrations of compound 2 and 3, or lower concentrations of compound 4. Interestingly, priming with the highest concentration (10^–4^ M) of compound 4 caused strong growth inhibition, leading to seedlings reaching only half the size of the control (MOCK) seedlings (Figure 3 and Supplementary Figure 1).

The rosette areas of the seedlings were further analyzed twice a day for an additional 6 days to record a growth curve (Supplementary Figure 2). All four compounds improved Arabidopsis seedling growth under control and stress conditions at some of the concentrations tested and there was significant interaction between compound concentration and growth conditions according to ANOVA. On the other hand, the highest concentration of compound 4 (10^–4^ M) showed inhibitory activity under all three of the conditions tested and the rosette areas were significantly reduced to 20, 60, and 48% of those in the non-primed control (MOCK) seedlings under, respectively, control, salt, and osmotic stress (Supplementary Figure 2).

**FIGURE 4:**
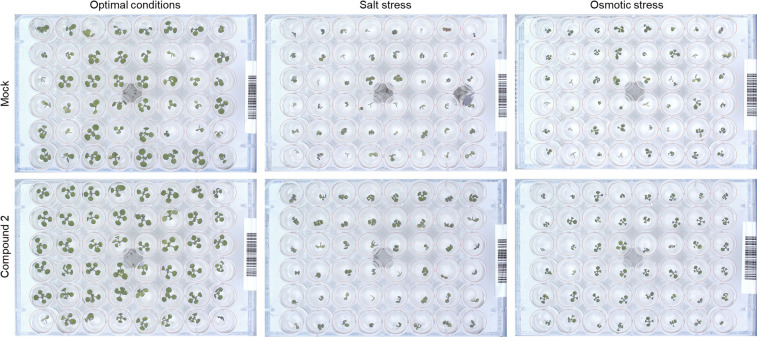
RGB images of the non-primed Arabidopsis seedlings (MOCK) and those primed with the best performing concentration of compound 2 (according to the PBC index, Table 3) grown under optimal growth condition (left images), salt (100 mM NaCl, middle images) or osmotic (100 mM mannitol, right images) stress.

Other traits such as relative growth rate (RGR) and absolute growth rate (AGR) were also calculated. For better visualization, these traits together with early seedling establishment and final rosette area (at day 7, Supplementary Figure 3) are presented in a parallel coordinate plot shown in Figure 3. To construct this, the differences between the controls and variants (compound and concentration) under the same growth conditions were calculated as the log2 of the ratio. The value obtained for each trait is shown in twelve independent parallel coordinate plots, one per compound (a total of 4) for optimal conditions, salt stress, and osmotic stress (Figure 3). Additionally, the priming effect of 10^–8^ BAP was evaluated as a positive control. Interestingly, in the parallel plot the three *N*^9^-substituted aromatic had similar profile whereas isoprenoid CK derivative showed different response (Figure 3). Under optimal growth conditions, priming with the new CK analogs improved some growth related traits analyzed (early seedling establishment, the slope of the curve, and AGR). At the assay end-point, mainly the seedlings primed with almost all concentrations of compound 2 had larger rosettes compared to the non-primed seedlings (MOCK) or those primed with the positive control (BAP) (Figure 3). These plants also presented higher homogeneity of the population (represented by coefficient of variance = standard deviation/Mean, %) compared with the MOCK variant (28.81 and 38.40, respectively) (Figure 4).

Importantly, hormopriming improved the tolerance of the Arabidopsis seedlings to salt and mannitol induced stress by increasing the values of the slope of the curve, RGR, ARG and final rosette size compared to the negative and positive controls (Figure 3). In both cases, low concentrations of compound 4 (10^–7^ M for salt stress and 10^–6^ M for osmotic stress) resulted in the highest increases in the traits analyzed, whereas a concentration of 10^–4^ M inhibited plant growth under all growth conditions (Figure 3 and Supplementary Figures 1, 2). Conversely, plants primed with compound 2 showed improvements in all traits under both control and stress conditions (Figure 4). All these results were then combined to calculate the PBC index, which helps to simplify and sum up the overall outcomes in order to define the mode of action of a biostimulant (Ugena et al., 2018). As listed in Table 3, all compounds worked as plant growth promotors and stress alleviators at some of the concentrations tested, all with higher efficiency than the control CK BAP. The most efficient plant growth promotor was compound 4 followed by compound 2. However, whereas compound 2 improved growth at all concentrations tested and growth conditions (working as strong plant growth promotor and stress alleviator), compound 4 was highly toxic at the highest concentration (10^–4^ M), at which it showed a growth inhibitory effect (Table 3). Overall, we conclude that priming with the newly prepared *N*^6^-substituted-2′-deoxy-2′-fluoro-9-(β)-D-arabinofuranosylpurines had positive effects on Arabidopsis growth and, importantly, improved tolerance to salt and osmotic stress, with a stronger effect in the latter case (Figures 3, 4 and Table 3).

**TABLE 3:**
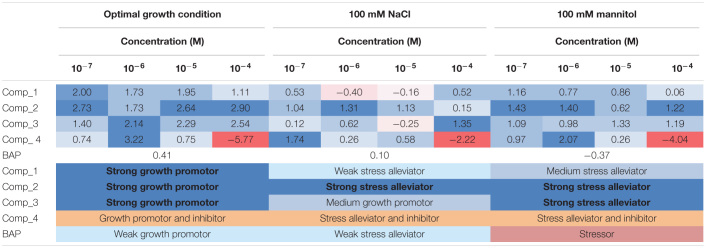
Plant biostimulant characterization (PBC) index calculated by summing the relative changes (log2) obtained for the parallel coordinate plot (Figure 4) for each synthetized compound (four different *N*^9^-substituted CK derivatives with a fluorinated carbohydrate moiety) at four concentration (10^–7^, 10^–6^, 10^–5^, and 10^–4^ M) and growth condition; optimal, salt stress (100 mM NaCl), or osmotic stress (100 mM mannitol) (*N* = 48).

*BAP at 10^–8^ M was used as positive control. Bold terms indicate the best performing compounds at the indicated growth conditions. Blue shades indicates positive effect, white no effect and red shade indicates negative effect.*

### Hormopriming With *N*^6^-Substituted-2′-Deoxy-2′-Fluoro-9-(β)-D-Arabinofuranosylpurines Maintains Seedling Greenness

**FIGURE 5:**
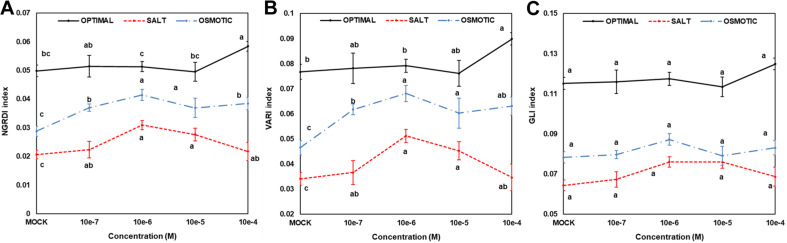
Color indices [NGRDI **(A)**, VARI **(B)**, or GLI **(C)**] used as greenness parameters for Arabidopsis seedlings from non-primed (MOCK) seeds or seeds primed with compound 2 at four concentrations (10^–7^, 10^–6^, 10^–5^, and 10^–4^ M) grown under optimal (upper panel), or salt (100 mM NaCl, middle panel), or osmotic (100 mM mannitol, bottom panel) stress conditions for 7 days (*N* = 48). Different letters mean significant differences among treatments (priming effect) for each growth conditions according to Dunn’s test after Kruskal–Wallis’ test.

To gain a further understanding of priming with compound 2 (*N*^6^-substituted-2′-deoxy-2′-fluoro-9-(β)-D-arabinofuranosylpurines), changes in seedling color after 7 days under different growth conditions (optimal, salt, or osmotic stress) were determined. The degradation of chlorophyll, manifested as a change in Arabidopsis rosette color, represents one of the most important symptoms of stress (Ugena et al., 2018). Three different indices (NGRDI, VARI, and GLI) were calculated and presented in Figure 5. Significant differences were observed between seedlings from non-primed and primed seeds, especially regarding NGRDI and VARI indices under all growth conditions (Figure 4). Under optimal conditions, the highest values were obtained when the compound 2 was applied at 10^–4^ M, a concentration that also resulted in the highest PBC index (Table 3). However, under salt and osmotic stress, the highest NGRDI and VARI indices were observed when 10^–5^ M and 10^–6^ M were used (Figure 5). Taken together, these results corroborated the aforementioned antisenescence effect of this compound observed in the CK-like bioassays (Table 2).

### Hormopriming With *N*^6^-Substituted-2′-Deoxy-2′-Fluoro-9-(β)-D-Arabinofuranosylpurines Improves Arabidopsis Growth and Stress Tolerance by Altering the Hormonal Profile

**FIGURE 6:**
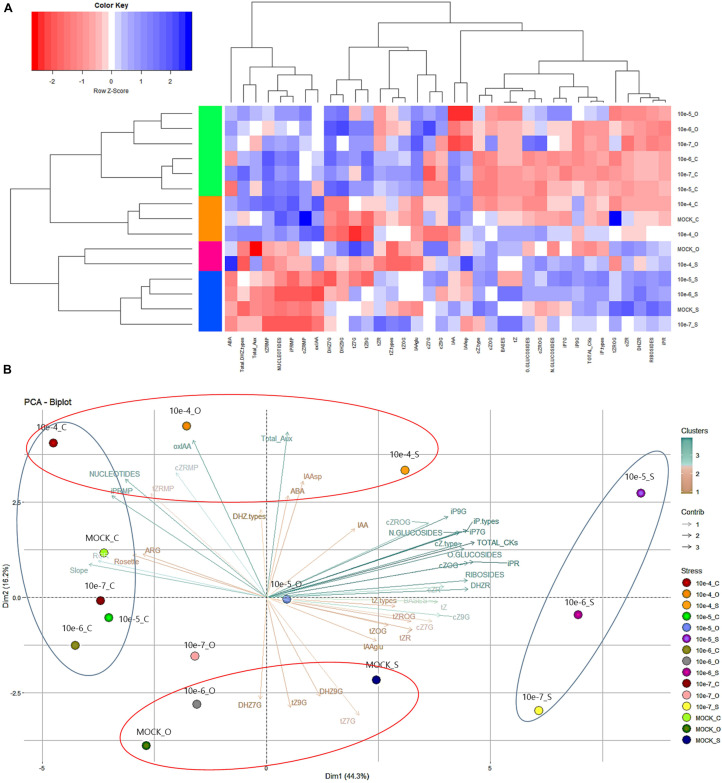
Heatmap **(A)** representing the changes in CKs, auxins, and ABA for Arabidopsis seedlings from non-primed (MOCK) seeds or seeds primed with compound 2 at four concentrations (10^–7^, 10^–6^, 10^–5^, and 10^–4^ M) grown under optimal (black line), or salt (100 mM NaCl, red line) or osmotic (100 mM mannitol, blue line) stress conditions for 7 days. Principal component analysis (Dimension, Dim) **(B)** of the same Arabidopsis seedlings.

To understand the molecular nature of the mode of action of priming by the *N*^6^-substituted-2′-deoxy-2′-fluoro-9-(β)-D-arabinofuranosylpurines, the hormonal profile of Arabidopsis seedlings primed with the best performing compound 2 was analyzed at the end of the phenotyping experiment. The endogenous levels of CKs (Table 4), some auxins and ABA were quantified using LC/MS (Supplementary Table 2). For better visualization and interpretation, all metabolites were analyzed together using a heatmap (Figure 6A). The results separated the variants (treatments and growth conditions) into two clusters using the Spearman correlation as the distance method: one for the plants grown under salt stress and the control for osmotic stress, and a second cluster for the rest. Additionally, the first group was separated into two subclusters in which all plants grown under salt stress showed, in general, a reduction in content of the CK nucleotides, total auxin, and ABA (Figure 6A). On the other hand, the variants represented in the second cluster showed increased levels of these metabolites and of some *N*- and *O*-glucosides (DHZ7G, DHZ9G, *t*Z7G, and *t*Z9G), and IAA conjugated with glucose (IAAGlu). On the other hand, they reduced content of total CKs, bases, and ribosides, especially in the case of the hormoprimed seedlings under optimal and osmotic stress conditions (Figure 6A). Similar results were obtained when the distance among variants was determined (Supplementary Figure 4), in which the hormopriming seedlings grown under optimal and osmotic stress were separated by a short distance (close to 0; similar behavior), but the distance was longer for the primed plants grown under salt stress conditions.

**TABLE 4:** Changes in CK levels (pmol g^–1^ FW) of 10-day-old *Arabidopsis thaliana* seedlings from non-primed seeds or seeds hormoprimed with compound 2 grown at four different concentrations (10^–7^, 10^–6^, 10^–5^, or 10^–4^ M) under optimal conditions, salt stress (100 mM NaCl), or osmotic stress (100 mM mannitol) for 7 days.

Conditions	Cytokinins	Optimal conditions				
		MOCK	10^–^^7^ M	10^–^^6^ M	10^–^^5^ M	10^–^^4^ M
Optimal conditions	Total CKs	104.73 ± 18.27	108.33 ± 14.54	97.85 ± 14.04	114 ± 21.56	114.54 ± 16.29
	Bases	0.08 ± 0.01	0.07 ± 0.02	0.08 ± 0.02	0.07 ± 0.02	0.07 ± 0.018
	Ribosides	18.65 ± 3.53	19.96 ± 4.08	16.98 ± 4.72	20.1 ± 5.28	16.77 ± 2.88
	Nucleotides	15.35 ± 5.90	12.25 ± 0.97	12.98 ± 1.44	14.73 ± 2.41	18.51 ± 3.29
	*O*-glucosides	7.53 ± 1.30	7.94 ± 1.21	6.65 ± 1.56	7.63 ± 2.02	6.49 ± 0.91
	*N*-glucosides	63.12 ± 9.19	68.11 ± 9.45	61.15 ± 7.43	71.46 ± 14.07	72.7 ± 9.93
	iP-types	47.40 ± 8.85	47.29 ± 5.40	45.92 ± 6.3	54.05 ± 9.58	56.09 ± 8.43
	iP	<LOD	<LOD	<LOD	<LOD	<LOD
	iPR	11.92 ± 2.64	12.3 ± 2.99	10.81 ± 3.08	13.3 ± 3.71	11.49 ± 2.23
	iPRMP	6.43 ± 1.43	6.05 ± 1.04	7.01 ± 0.62	7.23 ± 1.42	10.22 ± 1.72
	iP7G	25.02 ± 4.29	25.48 ± 3.05	24.99 ± 2.85	29.69 ± 5.79	29.84 ± 4.42
	iP9G	4.03 ± 0.68	3.46 ± 0.59	3.12 ± 0.39	3.83 ± 0.67	4.54 ± 0.49
	*t*Z-types	25.41 ± 4.52	32.79 ± 6.04	28.94 ± 3.01	32.33 ± 6.57	30.42 ± 6.23
	*t*Z	0.08 ± 0.01	0.07 ± 0.02	0.08 ± 0.023	0.07 ± 0.016	0.07 ± 0.018
	*t*ZR	2.27 ± 0.33	4.41 ± 1.32	4.07 ± 1.03	4.09 ± 0.89	3.06 ± 0.61
	*t*ZRMP	1.45 ± 0.25	2.1 ± 0.47	2.26 ± 0.22	2.12 ± 0.39	2.76 ± 0.65
	*t*ZOG	1.89 ± 0.33	2.92 ± 0.42	2.37 ± 0.54	2.94 ± 0.66	2.17 ± 0.37
	*t*ZROG	0.73 ± 0.20	0.32 ± 0.05	0.2 ± 0.03	0.35 ± 0.09	0.23 ± 0.02
	*t*Z7G	7.79 ± 1.82	10.17 ± 0.97	12.45 ± 2.59	13.53 ± 2.30	10.68 ± 2.86
	*t*Z9G	3.13 ± 0.61	9.79 ± 1.07	10.32 ± 2.66	8.6 ± 2.44	5.99 ± 1.37
	DHZ-types	2.96 ± 0.51	4.36 ± 0.74	3.46 ± 0.94	4.09 ± 0.90	4.42 ± 0.53
	DHZ	<LOD	<LOD	<LOD	<LOD	<LOD
	DHZR	0.15 ± 0.04	0.17 ± 0.07	0.12 ± 0.04	0.15 ± 0.05	0.09 ± 0.01
	DHZRMP	<LOD	<LOD	<LOD	<LOD	<LOD
	DHZOG	<LOD	<LOD	<LOD	<LOD	<LOD
	DHZROG	<LOD	<LOD	<LOD	<LOD	<LOD
	DHZ7G	1.5 ± 0.37	3.20 ± 0.90	3.80 ± 0.83	4.18 ± 0.48	1.49 ± 0.44
	DHZ9G	0.07 ± 0.02	0.13 ± 0.03	0.14 ± 0.03	0.17 ± 0.03	0.07 ± 0.02
	*c*Z-types	28.97 ± 7.73	23.89 ± 3.33	19.53 ± 4.8	23.52 ± 4.90	23.61 ± 2.97
	*c*Z	<LOD	<LOD	<LOD	<LOD	<LOD
	*c*ZR	4.33 ± 1.24	3.07 ± 0.47	1.98 ± 0.64	2.57 ± 0.66	2.16 ± 0.42
	*c*ZRMP	7.48 ± 5.00	4.1 ± 1.36	3.71 ± 1.04	5.37 ± 0.67	5.53 ± 1.02
	*c*ZOG	1.02 ± 0.22	0.58 ± 0.11	0.52 ± 0.1	0.59 ± 0.14	0.55 ± 0.13
	*c*ZROG	3.90 ± 0.86	4.12 ± 0.69	3.57 ± 1.02	3.76 ± 1.21	3.54 ± 0.77
	*c*Z7G	14.46 ± 3.81	9.16 ± 2.50	10.69 ± 2.64	11.32 ± 1.35	13.79 ± 3.38
	*c*Z9G	0.57 ± 0.14	0.59 ± 0.16	0.55 ± 0.14	0.51 ± 0.09	0.64 ± 0.20
Salt stress (100 mM NaCl)	Total CKs	157.34 ± 35.63	173.13 ± 27.9	208.79 ± 19.85	244.29 ± 61.86	164.18 ± 22.6
	Bases	0.13 ± 0.03	0.49 ± 0.09	0.46 ± 0.12	0.20 ± 0.05	0.20 ± 0.06
	Ribosides	75.35 ± 17.82	53.94 ± 16.41	76.5 ± 16.27	95.05 ± 28.28	44.51 ± 11.92
	Nucleotides	4.51 ± 1.06	4.25 ± 1.43	6.8 ± 0.96	9.36 ± 5.12	8.35 ± 2.36
	*O*-glucosides	8.48 ± 1.47	14.33 ± 3.63	15.66 ± 2.17	16.32 ± 3.80	14.5 ± 3.38
	*N*-glucosides	68.90 ± 17.88	100.11 ± 7.04	109.36 ± 13.89	123.37 ± 25.39	96.63 ± 18.40
	iP-types	67.33 ± 15.565	73.08 ± 7.08	95.7 ± 11.8	117.44 ± 25.00	80.28 ± 8.09
	iP	<LOD	<LOD	<LOD	<LOD	<LOD
	iPR	30.56 ± 5.83	27.47 ± 8.71	36.77 ± 10.13	47.19 ± 13.93	26.68 ± 8.57
	iPRMP	1.88 ± 0.49	1.7 ± 0.47	2.59 ± 0.56	3.12 ± 0.82	4.32 ± 0.51
	iP7G	30.49 ± 8.87	39.03 ± 5.18	51.16 ± 10.73	61.20 ± 9.55	45.16 ± 8.81
	iP9G	4.41 ± 1.09	4.88 ± 1.29	5.18 ± 0.19	5.93 ± 1.33	5.2 ± 1.42
	*t*Z-types	24.3 ± 6.30	57.99 ± 10.36	42.58 ± 9.15	41.62 ± 11.39	19.86 ± 4.25
	*t*Z	0.13 ± 0.03	0.49 ± 0.09	0.46 ± 0.12	0.20 ± 0.05	0.20 ± 0.06
	*t*ZR	4.19 ± 1.10	15.19 ± 4.67	7.14 ± 1.73	7.93 ± 2.54	2.25 ± 0.66
	*t*ZRMP	0.50 ± 0.12	0.43 ± 0.08	1.18 ± 0.26	1.73 ± 0.53	0.78 ± 0.18
	*t*ZOG	1.72 ± 0.51	5.5 ± 1.38	3.48 ± 0.97	3.65 ± 1.13	1.58 ± 0.44
	*t*ZROG	0.77 ± 0.21	0.7 ± 0.18	0.65 ± 0.13	0.68 ± 0.17	0.32 ± 0.11
	*t*Z7G	10.87 ± 2.14	19.13 ± 4.16	17.41 ± 4.46	9.97 ± 1.76	10.52 ± 2.24
	*t*Z9G	6.04 ± 1.36	10.54 ± 2.45	10.03 ± 2.59	4.76 ± 1.54	6.81 ± 1.89
	DHZ-types	2.04 ± 0.51	3.46 ± 0.81	4.06 ± 0.69	4.33 ± 1.06	2.22 ± 0.5
	DHZ	<LOD	<LOD	<LOD	<LOD	<LOD
	DHZR	0.58 ± 0.11	0.57 ± 0.12	1.02 ± 0.26	0.93 ± 0.25	0.32 ± 0.09
	DHZRMP	<LOD	<LOD	<LOD	<LOD	<LOD
	DHZOG	<LOD	<LOD	<LOD	<LOD	<LOD
	DHZROG	<LOD	<LOD	<LOD	<LOD	<LOD
	DHZ7G	2.59 ± 0.54	2.9 ± 0.54	3.27 ± 0.84	1.76 ± 0.52	2.65 ± 0.66
	DHZ9G	0.12 ± 0.01	0.14 ± 0.02	0.13 ± 0.03	0.13 ± 0.04	0.13 ± 0.04
	*c*Z-types	63.67 ± 15.175	38.6 ± 10.84	66.44 ± 9.76	80.9 ± 25.69	61.83 ± 10.99
	*c*Z	<LOD	<LOD	<LOD	<LOD	<LOD
	*c*ZR	40.02 ± 12.19	10.72 ± 3.1	31.58 ± 6.52	39 ± 12.4	15.25 ± 3.84
	*c*ZRMP	2.13 ± 0.55	2.12 ± 1.02	3.03 ± 0.57	4.51 ± 3.81	4.34 ± 1.22
	*c*ZOG	1.90 ± 0.59	2.02 ± 0.53	1.87 ± 0.19	3.08 ± 0.76	2.43 ± 0.75
	*c*ZROG	4.10 ± 0.90	6.12 ± 1.58	9.67 ± 1.38	8.91 ± 1.95	10.17 ± 2.52
	*c*Z7G	14.49 ± 2.53	19.28 ± 2.58	24.63 ± 7.6	28.43 ± 8.26	16.72 ± 4.28
	*c*Z9G	0.73 ± 0.17	1.03 ± 0.16	0.78 ± 0.25	1.21 ± 0.39	0.86 ± 0.28
Osmotic stress (100 mM mannitol)	Total CKs	83.87 ± 18.50	112.08 ± 8.93	103.89 ± 9.47	148.80 ± 15.57	138.51 ± 10.90
	Bases	0.056 ± 0.01	0.10 ± 0.02	0.10 ± 0.02	0.10 ± 0.03	0.23 ± 0.08
	Ribosides	16.83 ± 3.28	15.71 ± 2.19	13.91 ± 3.03	21.62 ± 2.88	25.41 ± 4.07
	Nucleotides	5.64 ± 1.52	10.41 ± 1.25	9.43 ± 1.44	15.15 ± 2.53	16.63 ± 0.98
	*O*-glucosides	6.44 ± 1.27	11.41 ± 1.34	8.71 ± 1.13	11.30 ± 1.15	12.15 ± 1.75
	*N*-glucosides	54.92 ± 14.04	74.45 ± 6.65	71.77 ± 6.45	100.63 ± 10.93	84.09 ± 10.49
	iP-types	41.23 ± 9.84	49.60 ± 2.20	46.33 ± 3.91	70.72 ± 6.57	61.58 ± 6.30
	iP	<LOD	<LOD	<LOD	<LOD	<LOD
	iPR	10.83 ± 2.35	11.25 ± 1.62	9.02 ± 1.97	15.12 ± 1.64	16.54 ± 2.72
	iPRMP	2.10 ± 0.63	4.93 ± 1.25	3.8 ± 0.75	6.81 ± 1.36	6.04 ± 1.26
	iP7G	25.38 ± 7.08	30.05 ± 1.13	30.24 ± 1.85	44.51 ± 4.67	35.41 ± 5.77
	iP9G	2.94 ± 0.82	3.38 ± 0.53	3.28 ± 0.72	4.28 ± 0.74	3.58 ± 0.33
	*t*Z-types	16.33 ± 3.34	28.25 ± 3.95	24.96 ± 2.68	31.01 ± 6.53	28.95 ± 3.19
	*t*Z	0.056 ± 0.01	0.10 ± 0.02	0.10 ± 0.02	0.10 ± 0.026	0.23 ± 0.08
	*t*ZR	2.04 ± 0.46	2.78 ± 0.61	2.29 ± 0.32	3.21 ± 0.69	4.15 ± 0.26
	*t*ZRMP	0.565 ± 0.15	1.47 ± 0.41	1.08 ± 0.27	1.66 ± 0.28	1.96 ± 0.38
	*t*ZOG	1.595 ± 0.31	2.69 ± 0.52	2.25 ± 0.44	3.18 ± 0.56	2.58 ± 0.36
	*t*ZROG	0.32 ± 0.05	0.41 ± 0.06	0.37 ± 0.07	0.33 ± 0.08	0.41 ± 0.11
	*t*Z7G	11.815 ± 2.56	11.59 ± 2.08	13.41 ± 2.63	11.21 ± 1.53	4.01 ± 1.07
	*t*Z9G	6.26 ± 1.51	7.3 ± 1.15	9.12 ± 2.94	8.42 ± 1.37	2.19 ± 0.65
	DHZ-types	2.08 ± 0.50	3.14 ± 0.24	3.32 ± 0.79	4.33 ± 1.08	4.58 ± 0.66
	DHZ	<LOD	<LOD	<LOD	<LOD	<LOD
	DHZR	0.15 ± 0.04	0.11 ± 0.03	0.07 ± 0	0.15 ± 0.05	0.22 ± 0.07
	DHZRMP	<LOD	<LOD	<LOD	<LOD	<LOD
	DHZOG	<LOD	<LOD	<LOD	<LOD	<LOD
	DHZROG	<LOD	<LOD	<LOD	<LOD	<LOD
	DHZ7G	1.92 ± 0.33	3.17 ± 0.76	4 ± 1.01	4.2 ± 0.67	1.06 ± 0.34
	DHZ9G	0.11 ± 0.03	0.11 ± 0.02	0.17 ± 0.05	0.17 ± 0.03	0.05 ± 0.01
	*c*Z-types	24.23 ± 5.10	31.09 ± 2.9	29.28 ± 2.93	42.74 ± 6.71	43.4 ± 1.98
	*c*Z	<LOD	<LOD	<LOD	<LOD	<LOD
	*c*ZR	3.83 ± 0.97	1.57 ± 0.17	2.56 ± 0.75	3.14 ± 0.91	4.5 ± 1.23
	*c*ZRMP	2.98 ± 0.935	4.01 ± 0.52	4.55 ± 0.63	6.68 ± 1.68	8.63 ± 1.23
	*c*ZOG	0.87 ± 0.19	1.4 ± 0.26	0.75 ± 0.16	0.96 ± 0.3	1.12 ± 0.25
	*c*ZROG	3.67 ± 0.835	6.91 ± 0.72	5.34 ± 0.99	6.84 ± 1.37	8.04 ± 1.15
	*c*Z7G	14.20 ± 2.68	15.53 ± 2.02	24.35 ± 4.67	20.14 ± 2.4	7.87 ± 2.07
	*c*Z9G	0.67 ± 0.18	0.55 ± 0.16	0.79 ± 0.23	0.96 ± 0.14	0.34 ± 0.07

*Mean and SD.*

To extend the analysis, a PC analysis was also performed (Figure 6B). The components PC1 and PC2 accounted for 60.5% of the total variance of the model. In PC1, there was clear evidence of contrasting behavior between all plants grown under optimal conditions and hormoprimed seedlings under salinity stress (blue ellipses). Thus, whereas the first group was positively correlated with the phenotyping traits and the CK nucleotides (synthesized *de novo*), which also showed a strong relationship (Supplementary Figure 5), the second group had a higher content of total CKs due to an increase in ribosides and *N*-glucosides (iP7G and iP9G) and *O*-Glucosides (*c*ZOG and *c*ZROG) (Figure 6B). Interestingly, hormopriming with the highest concentration (10^–4^ M) of compound 2 (as shown by PC2, red ellipses) induced similar contents of total auxins and the degradation form 2-oxindole-3-acetic acid (oxIAA) independent of growth conditions (Figure 6B), a pattern opposite to that in the MOCK variant under salt and osmotic stress. Overall, we demonstrated that in general hormopriming with the *N*^6^-substituted-2′-deoxy-2′-fluoro-9-(β)-D-arabinofuranosylpurines presented here induced changes in the hormonal content of Arabidopsis seedlings, thus conditioning the final phenotype, with the changes depending on the concentration of the compound and on growth conditions.

## Discussion

In this work, a group of four *N*^9^-substituted CK derivatives with a fluorinated carbohydrate moiety (three with an aromatic and one with isoprenoid *N*^6^ side chain) were synthesized (Wan et al., 2005) by a slightly modified one-step reaction of 9-(2′-deoxy-2′-fluoro-β-D-arabinofuranosyl)hypoxanthine with the appropriate amine or amine hydrochloride in the presence of BOP and DIPEA in DMF (Figure 1 and Table 1). Nucleosides bearing fluorine or fluorinated substituents within the carbohydrate moiety have been used successfully in many biochemical research studies and therapeutic treatments. As an example, the ability of 9-(2-deoxy-β-D-arabinofuranosyl)adenine to completely inhibit the protozoan parasite *Trichomonas vaginalis* (Shokar et al., 2012), as well as its antibacterial (Gao et al., 2015) and antitrypanosomal (Ranjbarian et al., 2017) effect, have been reported. Significant antiviral activity was also confirmed for their dideoxy analogs. Montgomery et al. (1992) proved that 2-fluoro-9-(2,3-dideoxy-2-fluoro-β-D-arabinofuranosyl)adenine had anti-HIV properties, and 9-(2,3-dideoxy-2-fluoro-β-D-arabinofuranosyl)adenine was identified as an anti-HBV agent (Maruyama et al., 1999). However, their effects on plant species have never been investigated. Only few works presenting positive bioassay results for kinetin and isopentenyladenine analogs with *N*^9^-substituted with short aliphatic chains been published (Mik et al., 2011a, b). Similar to these results, our newly synthesized compounds (especially compound 2) also showed high levels of antisenescence activity. Moreover, the low activity of the *N*^6^-substituted-2′-deoxy-2′-fluoro-9-(β)-D-arabinofuranosylpurine derivatives in the callus bioassay confirms that fluorination of the sugar moiety prevents its hydrolysis to free bases and makes these compounds metabolically stable. Due to the strong effect of the CK analogs with a fluorinated carbohydrate moiety on the retention of chlorophyll in excised wheat leaves in the dark (Table 2), we hypothesized that the compounds would have antistress properties and analyzed their mode of action and their potential use as priming agents.

For many years, it has been shown that seed priming with certain hormones or other compounds improves seed germination and fitness in many plants (Van Hulten et al., 2006; Hussain et al., 2016). Seed priming improves stress tolerance through “priming memory,” which is established during priming and can be recruited later when seeds are exposed to stresses during germination (Chen and Arora, 2013). The beneficial effect of seed priming with CKs has been previously described under a range of growth conditions for many plant species, such as spring wheat (*Triticum aestivum* L.) (Iqbal and Ashraf, 2005; Iqbal et al., 2006) or basil (*Ocimum basilicum* L) (Bagheri et al., 2014). Despite this, there is not always a clear positive effect of priming, and it may also have a negative effect (Miyoshi and Sato, 1997; Sneideris et al., 2015; Willams et al., 2016), depending on the type of compound, the concentration used for priming, or the plant species and cultivars tested (reviewed by De Diego and Spíchal, 2020). In this work, hormopriming with the new *N*^9^-substituted CK derivatives improved early seed establishment and plant growth in *A. thaliana* under optimal and stress growth conditions (Figure 3), mainly by making the population more homogeneous, maintaining plant greenness (less chlorophyll degradation) and better nutrient status as defined by higher color indices (Figure 5). However, this response was concentration dependent. The best-performing compound was 6-(3-hydroxybenzylamino)-2′-deoxy-2′-fluoro-9-(β)-D-arabinofuranosylpurine, which was a good growth promotor under optimal growth conditions and a stress alleviator under both salt and osmotic stress at almost all concentrations tested, according to the PBC index (Table 3). As an exception, compound 4 at 10^–4^ M showed a strong growth inhibitory (toxic) effect. However, lower concentrations (10^–7^ or 10^–6^ M) improved plant growth under different growth conditions (Table 3). This underlines the importance of testing chemicals over broad concentration ranges and under different growth conditions. This is possible through initial high-throughput approaches using model plants such as Arabidopsis, followed by studies in the targeted species and specific growth conditions (Rouphael et al., 2018).

To understand better how these new compounds modify plant metabolism when they are used as priming agents, the endogenous levels of some plant hormones (CK, auxins, and ABA) were quantified. It was clear that hormopriming with compound 2 disrupted the plants’ hormonal homeostasis (Figure 6B). However, the changes varied depending on the conditions under which the plants were grown. Thus, different behaviors were observed between hormoprimed seedlings under optimal and osmotic stress conditions, and those under salt stress (Supplementary Figure 4). For example, under optimal conditions, primed Arabidopsis seedlings accumulated higher levels of ribotides (precursors), which were positively correlated with phenotypic traits such as AGR, RGR, slope of the growing curve and final rosette area (Supplementary Figure 5A). However, primed plants grown under salt stress conditions elevated their total CK content by increasing the amounts of conjugated forms including ribosides (iPR. DHZR and *c*ZR), *O*-glucosides (*c*ZOG and *c*ZROG), and *N*-glucosides (iP7G and iP9G). It has been reported that riboside accumulation under stress conditions can be a defense mechanism, helping plants to deal with stress (Veerasamy et al., 2007; Man et al., 2011; De Diego et al., 2015). This may be because they play a crucial role in CK-mediated leaf longevity, and hence senescence, through phosphorylation of the CK response regulator ARR2 (reviewed by Hönig et al., 2018). For several years the *c*Z-type CKs and the base *c*Z were considered to be low-activity forms. However, in recent years, it has been proved that *c*Z-type CKs play important roles during plant development and in environmental interactions (Schäfer et al., 2015; Lacuesta et al., 2018). Thus, in primed Arabidopsis plants high levels of accumulation of *c*ZOG and *c*ZROG could be a strategy for maintaining plant growth under salt stress conditions. In support of this, it has been reported that the content of *c*Z-type CKs changes rapidly during maize seedling growth, and that *c*Z catabolism and glycosylation by *c*Z *O*-glucosyl transferases work synergistically to fine-tune *c*Z levels during plant development (Zalabák et al., 2014). Finally, in these primed plants there was also considerable accumulation of iP7G and iP9G. These two iP derivates are the terminal products of iP metabolism (Hošek et al., 2020). The iP metabolites including iP-N9G are the least active CKs, which seem not to be hydrolyzed and simply accumulate in the tissue (if not degraded by CKX) with no physiological effects (Hoyerová and Hošek, 2020). Overall, it is clear that priming with CK analogs modifies CK metabolism, but these changes are dependent on plant growth conditions. The results also pointed to the iP-type and *c*Z-type CKs as the main metabolites regulating the alleviation of salt stress in primed Arabidopsis seedlings.

Regarding auxins, levels of oxIAA mainly increased when compound 2 was applied at a high concentration (10^–4^ M) (Figure 6 and Supplementary Table 2). In recent years it has been proved that oxidizing IAA into oxIAA is of major physiological significance in the regulation of plant growth and development (Stepanova and Alonso, 2016). However, changes in other auxin-related metabolites did not show any correlation with the phenotypical changes in plants primed with compound 2.

## Conclusion

In summary, in this case study we showed that hormopriming with *N*^9^-substituted CK derivatives with a fluorinated carbohydrate moiety seems to be a promising biotechnological approach for improving early seedling establishment and plant growth under both control and stress conditions. This is due to changes in plant hormone metabolism (especially of CKs and auxins) that differs according to growth conditions. Moreover, we believe that we have shown here that a complex approach is needed for selection of suitable compounds, by employing strategies allowing simultaneous testing of a broad range of concentrations and different growth conditions to define the conditions in which they are most efficient as priming agents.

## Data Availability Statement

The original contributions presented in the study are included in the article/Supplementary Material, further inquiries can be directed to the corresponding author/s.

## Author Contributions

MB and KD synthesized the compounds. AH, AEH, LS, and ND designed and performed the phenotyping experiments. AH, AP, and ON carried out the metabolite quantification. AH and ND performed the data analysis. MB, AH, LS, KD, and ND wrote the manuscript. All authors discussed the results.

## Conflict of Interest

The authors declare that the research was conducted in the absence of any commercial or financial relationships that could be construed as a potential conflict of interest.

## Footnotes

1 http://www.plant-phenotyping.org/db_infrastructure#/tool/57

## Supplementary Material

Supplementary Figure 1 Early seedling establishment of Arabidopsis seedlings non-primed (MOCK) or primed with four different *N*^9^-substituted CK derivatives each with a fluorinated carbohydrate moiety at four concentrations (10^–7^, 10^–6^, 10^–5^, or 10^–4^ M) grown under control conditions (*N* = 48). Mean ± SE. Different letters mean significant differences among variants according to Tukey’s HSD test after ANOVA.

Supplementary Figure 2 Growth curves for Arabidopsis seedlings non-primed (MOCK) or primed with four different *N*^9^-substituted CK derivatives each with a fluorinated carbohydrate moiety at four concentrations (10^–7^, 10^–6^, 10^–5^, or 10^–4^ M) grown for 7 days under optimal, salt stress (100 mM NaCl), or osmotic stress (100 mM mannitol) conditions (*N* = 48). Mean ± SE.

Supplementary Figure 3 Maximum rosette size of Arabidopsis seedlings non-primed (MOCK) or primed with four different *N*^9^-substituted CK derivatives each with a fluorinated carbohydrate moiety at four concentrations (10^–7^, 10^–6^, 10^–5^, or 10^–4^ M) grown for 7 days under optimal, salt stress (100 mM NaCl), or osmotic stress (100 mM mannitol) conditions (*N* = 48). Mean ± SE. Different letters mean significant differences among variants according to Tukey’s HSD test after ANOVA.

Supplementary Figure 4 Distance between Arabidopsis seedlings non-primed (MOCK) or primed with compound 2 at four concentrations (10^–7^, 10^–6^, 10^–5^, or 10^–4^ M) grown for 7 days under optimal (C), salt stress (100 mM NaCl, S) or osmotic stress (100 mM mannitol, O) conditions.

Supplementary Figure 5 Correlation matrix **(A)** and contribution of the loadings to each PC (Dim) **(B)** according to multivariate statistical analyses of traits and metabolites in Arabidopsis seedlings non-primed (MOCK) or primed with compound 2 at four concentrations (10^–7^, 10^–6^, 10^–5^, or 10^–4^ M) grown for 7 days under optimal, salt stress (100 mM NaCl), or osmotic stress (100 mM mannitol) conditions (*N* = 48).

## Abbreviations

BAP: 6-benzylaminopurine
ABA: abscisic acid
CKs: cytokinins
IAA: indole-3-acetic acid
iP: *N*^6^-isopentenyladenine
*c*Z: *cis*-zeatin
DHZ: dihydrozeatin
*t*Z: *trans*-zeatin
HTS: high-throughput screening
PBC: plant biostimulant characterization index.
