# Supplementary figures and images for: Hormopriming to Mitigate Abiotic Stress Effects: A Case Study of N9-Substituted Cytokinin Derivatives With a Fluorinated Carbohydrate Moiety
Source: Front Plant Sci. 2020 Dec 10;11:599228. doi: 10.3389/fpls.2020.599228 (PMC7758400; doi:10.3389/fpls.2020.599228)

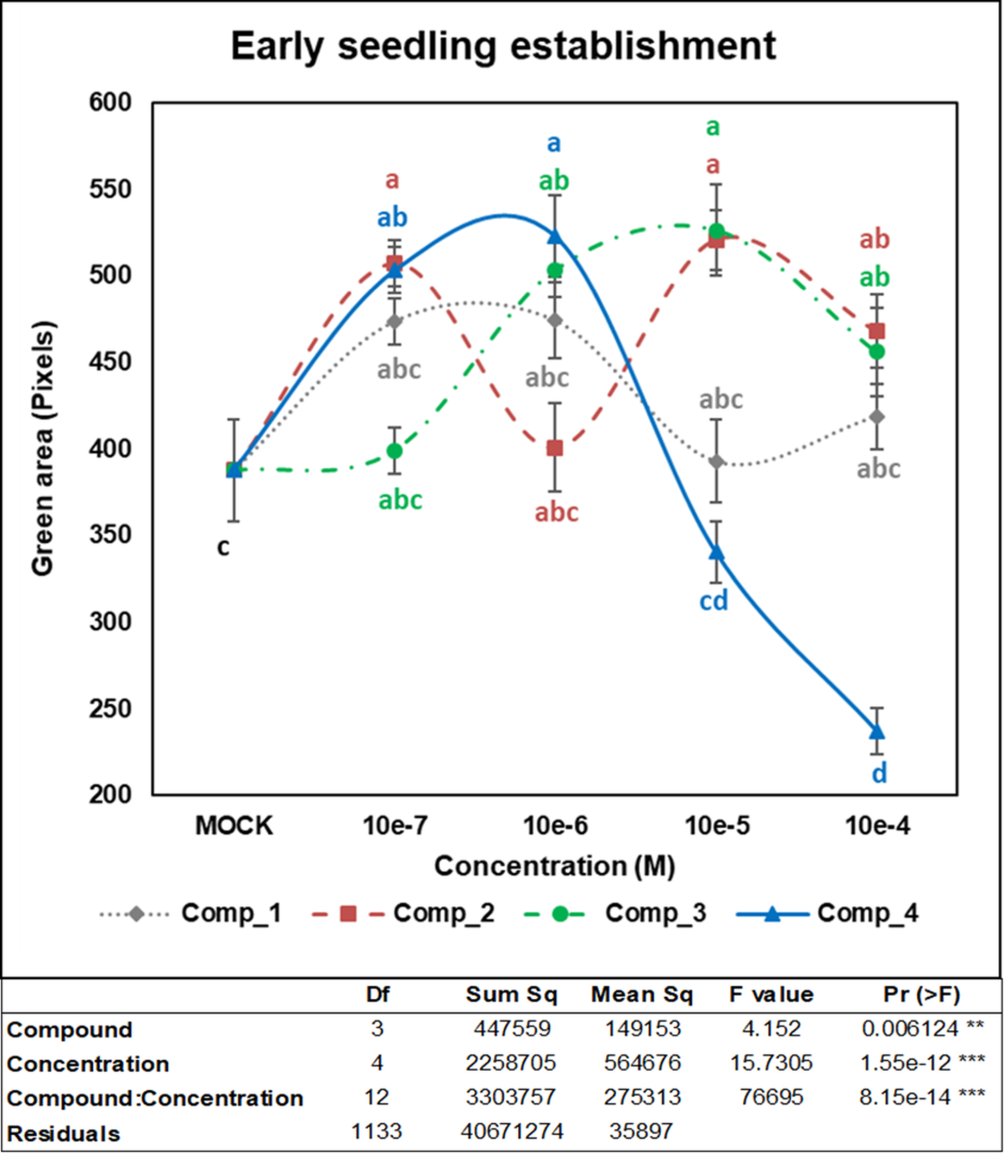

Supplement: Supplementary Figure 1 — Early seedling establishment of Arabidopsis seedlings non-primed (MOCK) or primed with four different N9-substituted CK derivatives each with a fluorinated carbohydrate moiety at four concentrations (10–7, 10–6, 10–5, or 10–4 M) grown under control conditions (N = 48). Mean ± SE. Different letters mean significant differences among variants according to Tukey’s HSD test after ANOVA. [file Image_1.TIF]

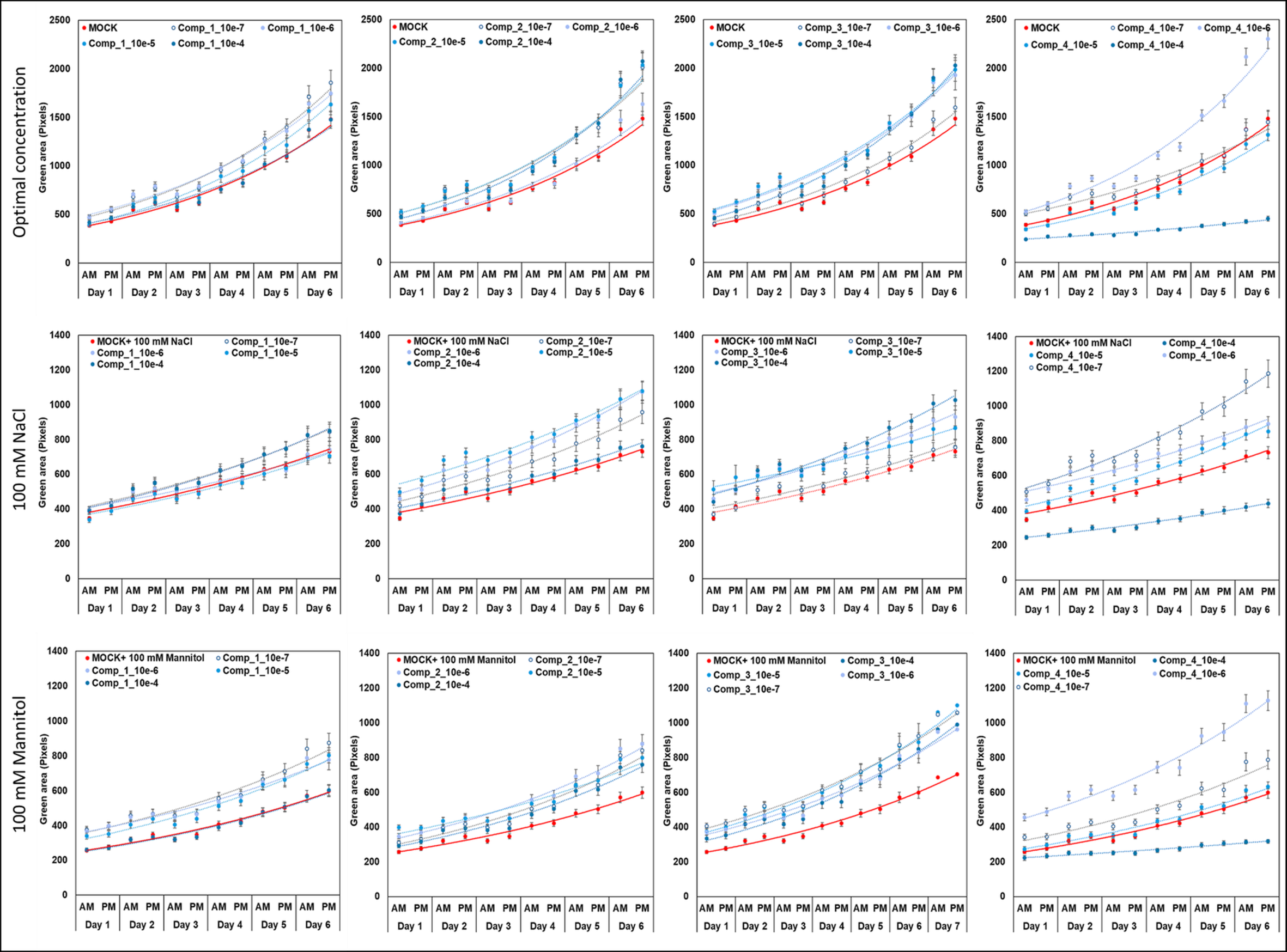

Supplement: Supplementary Figure 2 — Growth curves for Arabidopsis seedlings non-primed (MOCK) or primed with four different N9-substituted CK derivatives each with a fluorinated carbohydrate moiety at four concentrations (10–7, 10–6, 10–5, or 10–4 M) grown for 7 days under optimal, salt stress (100 mM NaCl), or osmotic stress (100 mM mannitol) conditions (N = 48). Mean ± SE. [file Image_2.TIF]

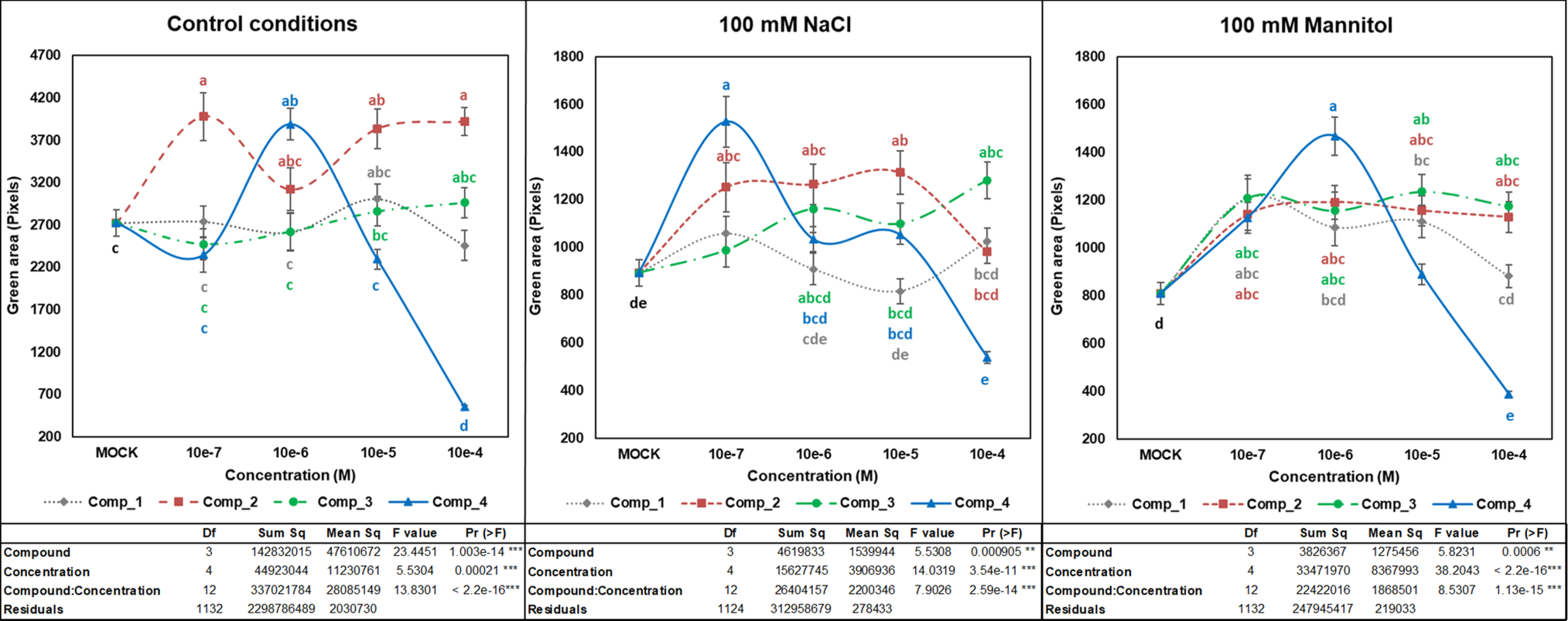

Supplement: Supplementary Figure 3 — Maximum rosette size of Arabidopsis seedlings non-primed (MOCK) or primed with four different N9-substituted CK derivatives each with a fluorinated carbohydrate moiety at four concentrations (10–7, 10–6, 10–5, or 10–4 M) grown for 7 days under optimal, salt stress (100 mM NaCl), or osmotic stress (100 mM mannitol) conditions (N = 48). Mean ± SE. Different letters mean significant differences among variants according to Tukey’s HSD test after ANOVA. [file Image_3.TIF]

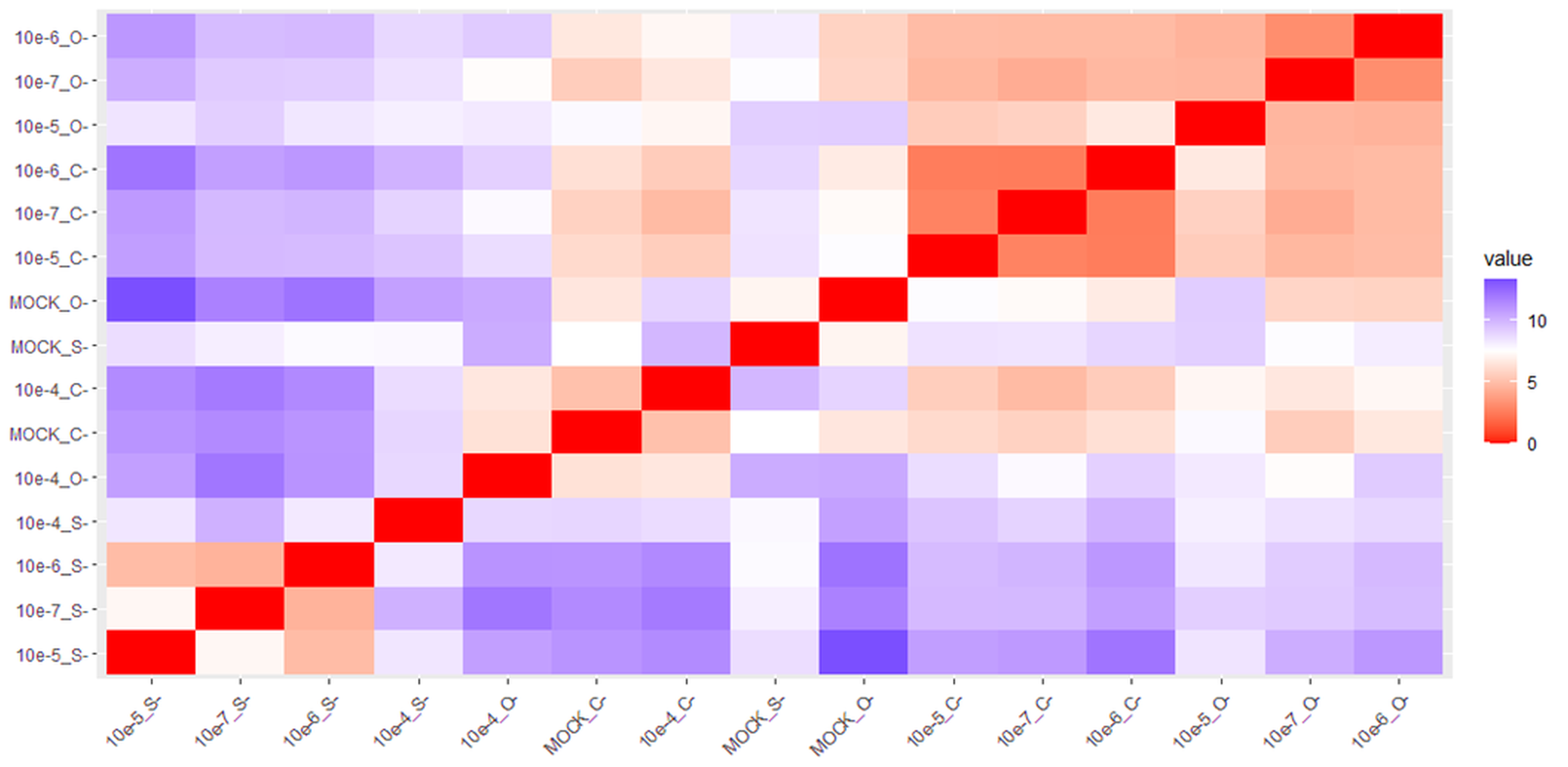

Supplement: Supplementary Figure 4 — Distance between Arabidopsis seedlings non-primed (MOCK) or primed with compound 2 at four concentrations (10–7, 10–6, 10–5, or 10–4 M) grown for 7 days under optimal (C), salt stress (100 mM NaCl, S) or osmotic stress (100 mM mannitol, O) conditions. [file Image_4.TIF]

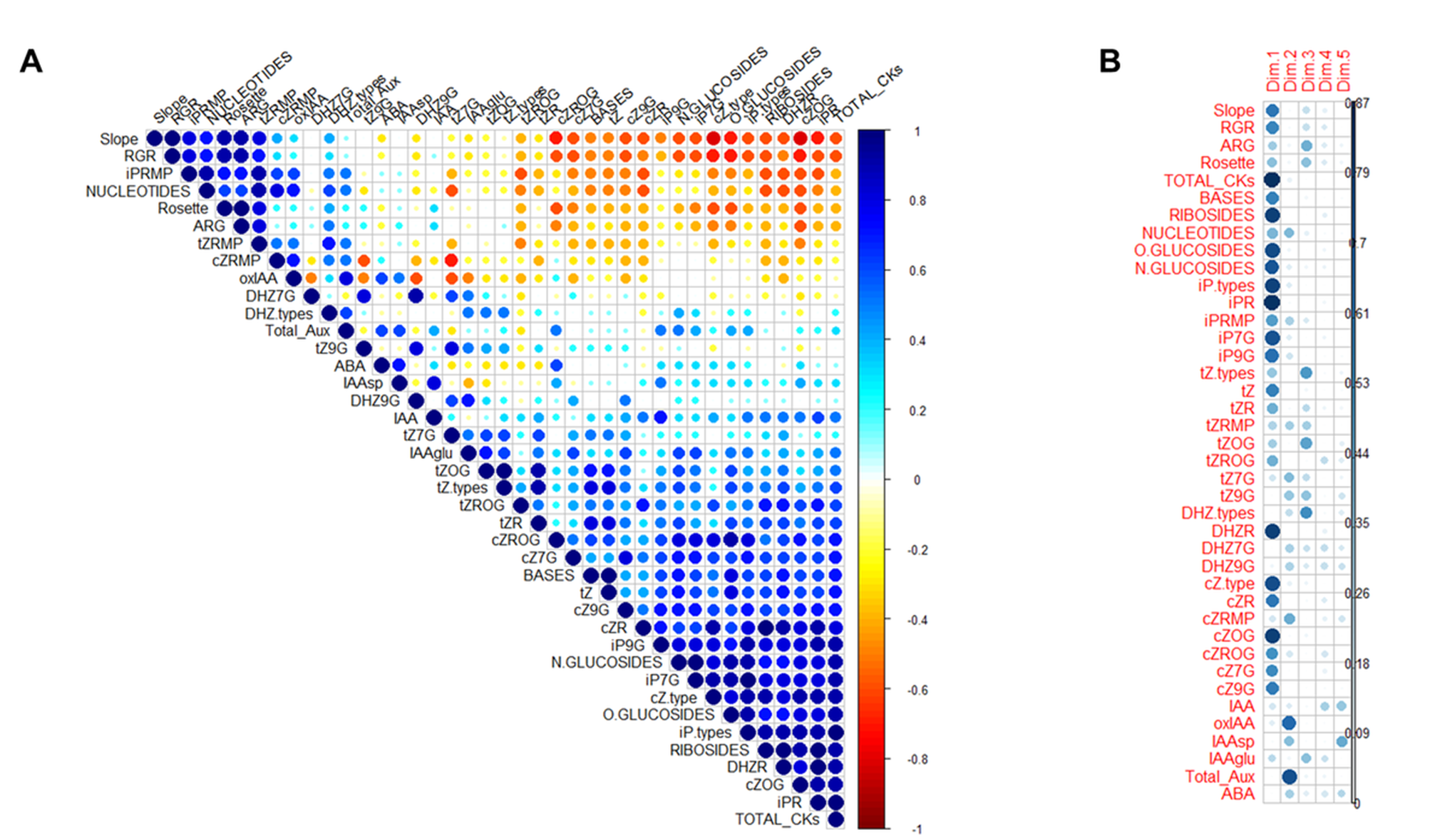

Supplement: Supplementary Figure 5 — Correlation matrix (A) and contribution of the loadings to each PC (Dim) (B) according to multivariate statistical analyses of traits and metabolites in Arabidopsis seedlings non-primed (MOCK) or primed with compound 2 at four concentrations (10–7, 10–6, 10–5, or 10–4 M) grown for 7 days under optimal, salt stress (100 mM NaCl), or osmotic stress (100 mM mannitol) conditions (N = 48). [file Image_5.TIF]
