# Supplementary material for: Hormopriming to Mitigate Abiotic Stress Effects: A Case Study of N9-Substituted Cytokinin Derivatives With a Fluorinated Carbohydrate Moiety
Source: Front Plant Sci. 2020 Dec 10;11:599228. doi: 10.3389/fpls.2020.599228 (PMC7758400; doi:10.3389/fpls.2020.599228)
Supplement: Supplementary file 6 [file Data_Sheet_1.PDF]

Supplementary Table S1|. Relative CK activities of prepared compounds in two classical CK bioassays. The optimal concentration for compounds **1-3** was compared with the activity of benzylaminopurine (BAP), where 100% means 10^{-5} M BAP. The optimal concentration for compound **4** was compared with the activity of isopentenyladenine (iP), where 100% means 10^{-5} M iP.

**Compound**	***Amaranthus* bioassay**		**Tobacco callus bioassay**	
	**Optimal concentration (M)**	**Relative activity (%)**	**Optimal concentration (M)**	**Relative activity (%)**
**1**	10^{-4}	72(±11)	10^{-5}	45(±2)
**2**	10^{-4}	15(±2)	10^{-5}	19(±1)
**3**	10^{-4}	37(±9)	10^{-5}	42(±3)
**4**	10^{-4}	22(±3)	10^{-7}	32(±9)
