# Supplementary material for: Hormopriming to Mitigate Abiotic Stress Effects: A Case Study of N9-Substituted Cytokinin Derivatives With a Fluorinated Carbohydrate Moiety
Source: Front Plant Sci. 2020 Dec 10;11:599228. doi: 10.3389/fpls.2020.599228 (PMC7758400; doi:10.3389/fpls.2020.599228)
Supplement: Supplementary file 7 [file Data_Sheet_2.PDF]

**Supplementary Table S2|** Changes in levels of certain auxins and ABA levels (pmol $g^{-1}$ FW) of 10-day-old *Arabidopsis thaliana* seedlings from non-primed seeds or seeds hormoprimed with compound 2 at four different concentrations ($10^{-7}$, $10^{-6}$, $10^{-5}$ or $10^{-4}$ M) grown under optimal conditions, salt stress (100 mM NaCl) or osmotic stress (100 mM mannitol) for 7 days. Mean and S.D.

| pmol $g^{-1}$ FW | Optimal conditions | | | | |
|---|---|---|---|---|---|
| | MOCK | $10^{-7}$ M | $10^{-6}$ M | $10^{-5}$ M | $10^{-4}$ M |
| IAA | 37.74 ± 11.47 | 43.47 ± 7.97 | 36.638 ± 9.63 | 41.381 ± 8.59 | 54.2 ± 20.828 |
| oxIAA | 193.35 ± 6.92 | 185.33 ± 14.10 | 169.72 ± 31.62 | 157.65 ± 29.64 | 360.8 ± 43.633 |
| IAAsp | 28.07 ± 4.07 | 28.38 ± 1.64 | 28.489 ± 4.25 | 31.29 ± 0.55 | 35.64 ± 4.6948 |
| IAGlu | <LOD | 62.11 ± 15.77 | 72.80 ± 11.53 | 50.79 ± 12.11 | <LOD |
| Total auxins | 243.09 ± 9.02 | 304.28 ± 32.54 | 291.28 ± 2.35 | 265.73 ± 9.87 | 430.4 ± 113.04 |
| ABA | 18.75 ± 4.20 | 26.18 ± 8.77 | 14.32 ± 4.01 | 13.44 ± 2.97 | 26.06 ± 1.64 |

| pmol $g^{-1}$ FW | Salt stress | | | | |
|---|---|---|---|---|---|
| | MOCK | $10^{-7}$ M | $10^{-6}$ M | $10^{-5}$ M | $10^{-4}$ M |
| IAA | 46.411 ± 11.41 | 50.74 ± 5.14 | 42.59 ± 13.94 | 63.70 ± 18.59 | 76.60 ± 6.11 |
| oxIAA | 126.15 ± 23.31 | 115.71 ± 1.28 | 116.4 ± 20.58 | 147.77 ± 21.69 | 314.01 ± 178.42 |
| IAAsp | 28.13 ± 1.763 | 24.73 ± 3.09 | 31.24 ± 16.27 | 30.02 ± 2.672 | 54.88 ± 24.16 |
| IAGlu | <LOD | 106.78 ± 16.63 | 92.47 ± 6.19 | 143.45 ± 14.68 | <LOD |
| Total auxins | 185.74 ± 30.45 | 287.13 ± 52.27 | 268.20 ± 33.92 | 368.59 ± 58.39 | 405.97 ± 221.97 |
| ABA | 15.40 ± 3.888 | 15.95 ± 1.44 | 19.89 ± 0.147 | 18.31 ± 2.35 | 61.79 ± 33.90 |

| pmol $g^{-1}$ FW | Osmotic stress | | | | |
|---|---|---|---|---|---|
| | MOCK | $10^{-7}$ M | $10^{-6}$ M | $10^{-5}$ M | $10^{-4}$ M |
| IAA | <LOD | <LOD | <LOD | <LOD | <LOD |
| oxIAA | 120.82 ± 26.70 | 164.46 ± 3.55 | 166.10 ± 47.80 | 194.59 ± 41.93 | 400.68 ± 97.79 |
| IAAsp | 20.45 ± 2.53 | 20.18 ± 1.41 | 21.74 ± 3.37 | 18.80 ± 1.55 | 29.18 ± 0.83 |
| IAGlu | <LOD | 71.42 ± 7.24 | 84.35 ± 14.87 | 141.42 ± 0.00 | <LOD |
| Total auxins | 129.78 ± 25.1 | 245.26 ± 18.49 | 259.64 ± 75.64 | 273.93 ± 52.89 | 414.86 ± 118.26 |
| ABA | 14.95 ± 1.03 | 21.03 ± 0.28 | 24.50 ± 4.85 | 26.87 ± 4.88 | 29.11 ± 3.13 |
